# A narrative overview of utilizing biomaterials to recapitulate the salient regenerative features of dental-derived mesenchymal stem cells

**DOI:** 10.1038/s41368-021-00126-4

**Published:** 2021-06-30

**Authors:** Sevda Pouraghaei Sevari, Sahar Ansari, Alireza Moshaverinia

**Affiliations:** 1 Weintraub Center for Reconstructive Biotechnology, Division of Advanced Prosthodontics, School of Dentistry, University of California, Los Angeles, Los Angeles, CA USA; 2 California NanoSystems Institute, University of California, Los Angeles, Los Angeles, CA USA

**Keywords:** Biotechnology, Mesenchymal stem cells

## Abstract

Tissue engineering approaches have emerged recently to circumvent many limitations associated with current clinical practices. This elegant approach utilizes a natural/synthetic biomaterial with optimized physiomechanical properties to serve as a vehicle for delivery of exogenous stem cells and bioactive factors or induce local recruitment of endogenous cells for in situ tissue regeneration. Inspired by the natural microenvironment, biomaterials could act as a biomimetic three-dimensional (3D) structure to help the cells establish their natural interactions. Such a strategy should not only employ a biocompatible biomaterial to induce new tissue formation but also benefit from an easily accessible and abundant source of stem cells with potent tissue regenerative potential. The human teeth and oral cavity harbor various populations of mesenchymal stem cells (MSCs) with self-renewing and multilineage differentiation capabilities. In the current review article, we seek to highlight recent progress and future opportunities in dental MSC-mediated therapeutic strategies for tissue regeneration using two possible approaches, cell transplantation and cell homing. Altogether, this paper develops a general picture of current innovative strategies to employ dental-derived MSCs combined with biomaterials and bioactive factors for regenerating the lost or defective tissues and offers information regarding the available scientific data and possible applications.

## Introduction

Reconstructing a lost or defective tissue often requires the challenging tasks of harvesting and grafting, which are associated with serious complications including pain, morbidity, and risk of infection. The need for less invasive alternatives has prompted scientists to focus on harnessing the regenerative potential of the human body to recreate the necessary architecture and function of lost or defective tissues.^1^ This need is particularly serious in cases of major traumatic defects such as bone loss where the human body’s self-repair mechanisms fail.^2^

Tissue engineering has emerged as a major multidisciplinary field that seeks to marry the benefits of life sciences with engineering principles to repair, regenerate, or enhance the function of defective tissues.^3^ The success of a tissue engineering approach depends on the appropriate selection of scaffolding material, stem cell type, and bioactive factors.^4^ An ideal biomaterial must be biocompatible, provide gas and nutrient exchange, protect cells from immune system invasion and external stresses, and provide suitable crosstalk between the encapsulated MSCs and the neighboring cells.^5^

Stem cells are indispensable for tissue development and regeneration. Their unique properties include self-renewal and multilineage differentiation capacity. The ability to produce stem cells in the large quantities required for the creation of macro-scale cell banks is central to the success of tissue engineering and personalized medicine.^6^ Additionally, the cells should be easily accessible with minimally invasive procedures and capable of maintaining their phenotype and stemness over time.^7^ The ethical concern in the case of embryonic stem cells (ESCs) is another factor limiting the application of certain types of stem cells.^8^ Human teeth and the surrounding tissue are unique treasures as they represent an easily accessible source of stem cells with few or no ethical issues.^9^ Biomaterials can serve as a promising platform for delivery of ex vivo cultured stem cells, or as an artificial niche facilitating homing of the local cells. In the current review paper, we seek to investigate how engineering principles combined with life sciences and biology can help scientists to harness the regenerative potential of dental stem cells for the regeneration of defective tissues. It is noteworthy to state that this review article will provide a general overview, and readers are encouraged to refer to more specific literature reviews for a more detailed discussion.

## Dental tissue-derived MSCs

**Fig. 1:**
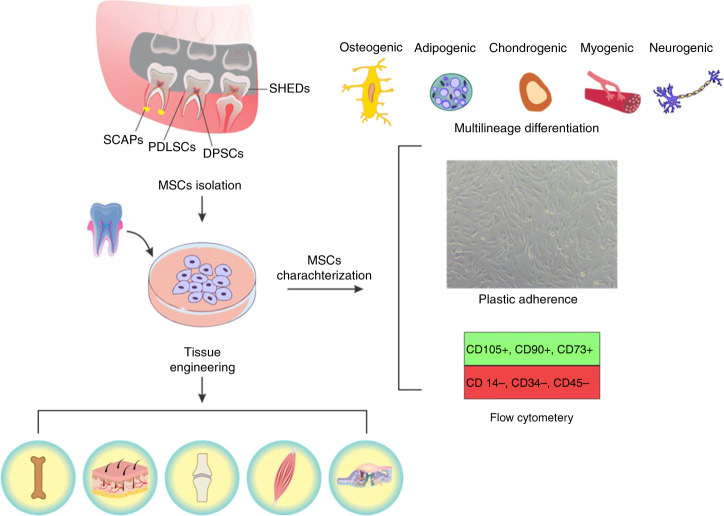
Mesenchymal stem cells (MSCs) residing in the oral cavity are harvestable from dental and associated tissues including stem cells from human exfoliated deciduous teeth (SHEDs), dental pulp stem cells (DPSCs), periodontal ligament stem cells (PDLSCs), and stem cells from the apical papilla (SCAPs). The minimal criteria to identify them include expression of MSC-specific surface markers, such as CD105, CD73, and CD90 but not CD45, CD34, or CD14; plastic adherence; and the ability to differentiate into multiple cell lineages including osteoblasts, adipocytes, chondroblasts, myocyte, and neurons. The harvested dental MSCs could be expanded ex vivo and utilized for regeneration of a wide range of tissues including bone, skin, cartilage, muscle, and sensory cells

Interest in stem cells within the oral cavity started with the discovery of adult stem cells and their potential to regenerate numerous tissue types. Mesenchymal stem cells (MSCs) are promising adult stem cells with multipotent and self-renewing potential that are obtainable from various tissues and capable of regenerating a wide range of impaired tissues.^10^ The minimal criteria to identify MSCs include plastic adherence; expression of MSC-specific surface markers, such as CD105, CD73, and CD90 but not CD45, CD34, or CD14 in over 95% of the population; and the ability to differentiate into multiple lineages such as osteoblasts, adipocytes, chondroblasts, myocyte, and neurons (Fig. 1).^11^

Bone marrow mesenchymal stem cells (BMMSCs) are a widely studied class of MSCs with potent regeneration capacity.^12^ However, BMMSCs suffer from serious issues such as invasive isolation procedures required to harvest them, the relatively low amount of available cells, and rapid cellular senescence and replicative exhaustion which limit their clinical applications.^13^ To circumvent these shortcomings, the fastest-growing biotechnology and life science sectors have discovered a class of MSCs residing in human teeth and dental tissue, often easily accessible, with immunoregulatory properties and differentiation capacity comparable to BMMSCs but superior growth potential.^14,15^ This discovery can pave the way for creating MSC banks at macro-scale levels from easily accessible cell sources with less invasive procedures and fewer ethical concerns.

Multipotent dental MSCs include, but are not limited to, stem cells obtained from dental pulp (DPSCs), exfoliated deciduous teeth (SHED), apical papilla (SCAPs), periodontal ligament (PDLSCs), and gingival tissue (GMSCs) (Fig. 1).^15,16^ These MSCs can contribute to the regeneration of dental and nondental tissues, such as muscle, bone, nerve, cartilage as shown in Fig. 1.^17–19^ They can be harvested from healthy or inflamed tissues or even biological waste that clinics would otherwise discard.^20^

The dental pulp is a valuable source of MSCs obtainable from exfoliated deciduous teeth (SHED), permanent teeth (DPSCs), extracted impacted teeth, teeth lost due to severe periodontitis, or inflamed dental pulp.^21^ It has been reported that transplantation of minced autologous pulp can contribute to pulp-dentin regeneration by locally supplying MSCs outgrown from the minced tissue.^22^

The dental pulp and apical papilla are continuous with one another; however, a question arises here regarding how an infected immature tooth with necrotic pulp could undergo root development and complete apexogenesis. This question led to the discovery of a unique type of infection-resistant stem cells originating from the apical papilla of immature permanent teeth (SCAP) with high rates of proliferation and self-renewal but low immunogenicity.^23^ Stem cells like these, originating from developing tissues, are known to possess advantages over those harvested from fully developed tissues. Originating from the pulp tissue, the SCAPs, DPSCs, and SHEDs are well-known for their versatile potential in developing odontoblast-like cells and pulp/dentin regeneration.^24^

The periodontium provides structural support to the tooth and harbors different types of MSCs. Dental follicle stem cells (DFCs) are one of these types, residing in the ectomesenchymal connective tissue loosely surrounding the developing tooth germ and possessing immunomodulatory and multipotent differentiation potential. They can give rise to the formation of cementum, periodontal ligament, and alveolar bone during tooth development.^25^ Extracted wisdom teeth are a valuable source from which to easily isolate DFCs for different tissue regeneration applications.^26^

The periodontal ligament (PDL) is an important component of the periodontium with fibers that extend toward the cementum and alveolar bone to attach the teeth to the surrounding tissue. This fibrous connective tissue is home to a class of dental MSCs named periodontal ligament stem cells (PDLSCs) that have multipotent and self-renewing properties. Although isolation of PDLSCs from the periodontal tissue can be invasive, an extracted premolar or wisdom tooth can be a good alternative from which to isolate them with less invasive procedures.^27^ It has been reported that about 27% of the cellular population residing in the human PDL are STRO-1^+^ PDLSCs.^28^

The scarless healing of gingival wounds prompted scientists to seek a population of stem cells responsible for this unique wound healing. The endeavor to understand the process led to the discovery of a new class of multipotent MSCs with profound immunoregulatory potential called gingival mesenchymal stem cells (GMSCs), which reside in the gingival tissue and are easily obtainable from patients’ gingival tissue with gingivectomy techniques or from biological waste tissue at dental clinics.^29^ Interestingly, GMSCs have higher proliferation rates than BMMSCs and can be isolated from healthy, hyperplastic, or inflamed tissue with similar morphology and karyotype.^30^

Dental-derived MSCs could be utilized for the regeneration of both dental and nondental tissues, as summarized in Table 1. The following sections will discuss some of the applications of dental MSCs in tissue engineering.

**Table 1:** Origin and therapeutic applications of dental MSCs

Type	Primary dentition	Permanent dentition			
	SHED	DPSC	SCAP	PDLSC	GMSC
Location	Exfoliated deciduous tooth	Permanent tooth pulp	Apical papilla of immature permanent tooth	Periodontal ligament	Gingival tissue
Tissue engineering	Bone Pulp/dentin Nerve	Bone Pulp/dentin Whole tooth regeneration Nerve Cartilage Muscle	Pulp/dentin Whole tooth regeneration Nerve	Periodontal tissue Whole tooth regeneration Nerve Cartilage	Gingival tissue Muscle Nerve Sensorineural hearing regeneration

## Dental MSCs and periodontal tissue engineering

**Fig. 2:**
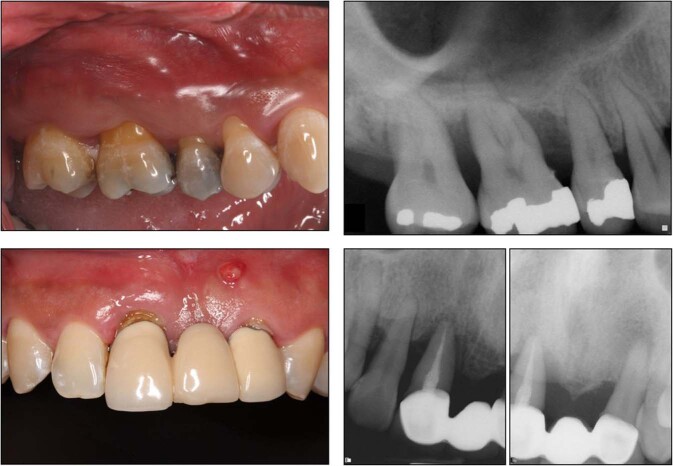
Clinical and periapical radiographic images of a patient with periodontal disease showing gingival inflammation and alveolar bone resorption

Periodontitis, a chronic inflammatory disease, is one of the most prevalent chronic infections in humans and leads to the destruction of the periodontium including alveolar bone, the periodontal ligament (PDL), and root cementum (Fig. 2).^31–33^ Different types of dental stem cells including PDLSCs and GMSCs have been studied as promising candidates for the regeneration of periodontal tissue and reconstruction of the bone-PDL complex. These cells can contribute to PDL tissue regeneration by secreting trophic and immunoregulatory factors to downregulate inflammation while regenerating the defective tissue.^34^ For instance, it has been reported that SHED can reduce gum bleeding, promote attachment of periodontal ligaments, and support periodontal tissue regeneration by regulating inflammation and infection as well as inducing M2 macrophage polarization and downregulating osteoclastogenic activity.^35^

Cell sheet engineering is a promising approach to delivering a large number of cells without disturbing cellular interactions. In one such study, a bilayered cell sheet composed of a layer of PDLSCs and a layer of osteoblasts was used to reconstruct the bone-PDL complex.^36^ Ectopic and orthotopic transplantation studies confirmed the ability of the engineered three-dimensional (3D) cell sheet to reconstruct the bone-PDL complex with functional PDL fiber attachments to the tooth root and alveolar bone.

Over the past decade, strategies for combatting periodontitis-induced bone loss have been mainly based on conventional anti-infectious measures, guided tissue regeneration (GTR), and the application of growth factors/bioactive molecules, but inconsistent results have underscored the need for more effective solutions.^37^ Tissue engineering approaches have emerged recently to circumvent many limitations associated with current clinical practices. These approaches utilize a natural or synthetic biomaterial with optimized physiomechanical properties, combined with stem cells and/or bioactive molecules to maintain the space for selective in-growth of the PDL and bone tissues while preventing or retarding the apical migration of the gingival epithelium.^38,39^ For such applications, a biomaterial should be biocompatible and have no risk of disease transmission, yet also biodegradable and porous to allow new tissue formation.

Nanofibrous membranes developed by electrospinning can produce highly porous structures capable of mimicking the natural extracellular matrix (ECM).^40,41^ Due to their small pore sizes, the cell penetration within the nanofibrous membranes is slow and thus they offer excellent structural and physical properties for GTR strategies, forming a barrier between two separate tissues without affecting their independent growth. Their microarchitecture and biodegradation can be tailored by adjusting the fabrication parameters to match the recovery kinetics of the damaged tissue.^42,43^ Furthermore, their surfaces can be modified to enhance the interfacial interactions with cells and surrounding tissues.

Polymeric periodontal membranes are among the promising alternatives. However, the existing membranes have low structural integrity, lack suitable mechanical strength, and bioactivity, and have a fast/uncontrolled degradation rate (in the case of absorbable membranes) or a second surgical procedure is necessary to remove the membrane (in the case of non-absorbable membranes).^44^ Therefore, there is an unmet need for a bioactive and biodegradable periodontal membrane capable of integrating well with the surrounding tissue for bone regeneration.

Fibrous membranes based on the FDA-approved polymer Poly(ε-caprolactone) (PCL) have received noticeable attention in this context due to their high biocompatibility and tunable properties necessary to support cellular growth and mineral deposition.^45,46^ In an interesting approach, multiple physicochemical techniques have been combined to develop a novel micropatterned PCL-based nanofibrous membrane coated with polydopamine (PDA) as a biomimetic niche for delivery of PDLSC aggregates for periodontal tissue regeneration.^46^ Inspired by the superior ability of mussels to adhere to wet surfaces, the presence of dopamine-based structures on the PCL membrane provides favorable adhesive properties, even in wet conditions in the presence of blood, saliva, and body fluids. Besides, it can accelerate the hydroxyapatite mineral deposition for improved bone regeneration.^46^ Also, PDA coatings appear to be an effective and inexpensive approach to produce a substrate with prolonged contact-active antibacterial function for the treatment of periodontitis-associated bone loss.^47,48^

## Dental MSCs and bone regeneration

Inspired by the natural bone remodeling processes, which require a well-organized harmony between osteoblasts and osteoclasts activity, regeneration of bone defects by directed differentiation of stem cells. The directed differentiation of stem cells could happen by providing necessary signalings through directly transplanting them within the defect site to receive the signals from the natural microenvironment or utilize biomaterials to provide the signals. These biomaterial-assisted signals could be applied in the form of an osteogenic scaffold, such as calcium phosphate-based materials or release of osteogenic growth factors.^49,50^

Despite the higher affinity of the BMMSCs in differentiating toward osteoblasts, certain complications associated with their harvesting methods and low cell yield have prompted scientists to actively look for alternative sources, which are abundant and easily accessible in this regard. Since osteoblasts originate from mesenchymal stem cells, the dental MSCs have gained significant attention over the past few years as an abundant favorite source for bone regeneration.^51^

A comparative study of the bone regenerative potential of human-derived DPSCs, SHEDs, and BMMSCs has reviled an equivalent regenerative potential among them; however, SHEDs could develop a larger osteoid area and the highest percentage of collagen fiber compared to the other two groups.^52^ Considering the less invasive harvesting procedure but equivalent bone regenerative potential, SHED could be a promising alternative to BMMSCs specifically for critical size bone defects.

Despite the abundance of studies, a diseased microenvironment such as inflamed tissue is a major obstacle in the clinical translation of stem cells because it not only devitalizes the regenerative potential of endogenous MSCs but it also affects the efficacy of the transplanted cells.^53^ For instance, the presence of inflammatory cytokines such as tumor necrosis factor-α (TNF-α) and interferon-γ (IFN-γ) along with the accumulation of reactive oxygen species (ROS) in the pathogenic microenvironment are among the factors that not only threaten the survival of the transplanted MSCs but also decreases the regenerative potential of the host tissue and consequently, causing an extended bone loss.^54,55^ Accordingly, the future strategies could target regenerating the pathogenic niche to restore the potential of endogenous stem cells and increasing the MSCs resistance to the pathogens by employing biomaterial-mediated therapies. In one such study, it has been reported that encapsulation of SHEDs within the alginate hydrogel could hinder the penetration of proinflammatory cytokines and increase their bone regenerative potential to a level comparable to BMMSCs by increasing cell survival rate.^56^

A combination of ex vivo expanded dental MSCs with osteogenic biomaterial followed by direct transplantation within the defect site has shown some success.^57,58^ For maximum survival, the transplanted cells need to have access to a source of oxygen and adapt their glucose consumption.^59^ However, lack of proper vascularization and impaired gas and nutrient exchange are among the major drawbacks hindering the successful translation of biomaterial-mediated bone regeneration approaches. Proper design of biomaterial with appropriate pore size and incorporation of angiogenic growth factors within the scaffolds could help to complement vascular in-growth from the host tissue into the grafted material and secure adequate gas and nutrient exchange.^60,61^

## Dental MSCs and whole tooth regeneration

Dental implants have served as the gold standard intervention for replacing the lost tooth for many years. Despite the fact that these implants are successfully serving the purpose in many cases, the impediments associated with their application such as infection and implant failure are raising significant concerns.^62^ Therefore, scientists are seeking alternative approaches to replace dental implants with a living replacement tooth by benefiting from the inherent revitalizing potential of the tooth for bio-root engineering and whole tooth regeneration.^63^

In this context, the bioengineering approaches could provide us with the necessary toolbox to recapitulate the regenerative potential of the dental MSCs.^64^ These de novo regenerated teeth could be developed by seeding the dental MSCs within a decellularized tooth scaffold or a tooth-shaped material combined with necessary growth factors.^65^ For instance, decellularized porcine tooth buds seeded with porcine dental epithelial cells or human DPSCs have shown the successful formation of a bioengineered tooth with organized dentin and enamel-like tissues in a mini-pig animal model.^66^

To circumvent the difficulties associated with decellularized natural tissues, artificially synthesized biomaterials, such as gelatin methacrylate (GelMA) hydrogel or poly(glycolic acid) (PGA)/poly(lactic-co-glycolic acid) (PLGA) copolymers, could be employed as a base for seeding the dental stem cells for regeneration of tooth buds.^67,68^ In another attempt, a bio-root periodontal complex capable of supporting a porcelain crown was developed by mixing SCAPs and PDLSc with hydroxyapatite/tricalcium phosphate (HA/TCP) particles in a swine animal model.^69^

Besides the incorporation of ex vivo expanded MSCs, cell homing approaches could be combined with biomaterials to recruit the host cells for regeneration of anatomically shaped tooth. In this approach, growth factors such as SDF-1 and BMP-7 can be incorporated within the tooth-shaped scaffolds to call the adjacent MSCs to action.^70^

## Dental MSCs for nondental tissue regeneration

As has already been mentioned, in addition to their ability to regenerate the orofacial tissues, dental-derived MSCs are capable of regenerating nondental tissues when the necessary microenvironment is given. PDLSCs encapsulated within RGD-coupled alginate microspheres impregnated with TGF-β1 have shown cartilage differentiation capability, as confirmed by the expression of chondrogenic markers, such as Col II and Sox9.^71^ Due to their potential to differentiate toward mesodermal lineages such as chondrocytes, DPSCs are another promising cell source for cartilage regeneration that can be directly administered to cartilage defect sites such as the knee with intra-articular injections in a minimally invasive manner.^72,73^ Additionally, superior angiogenic and regenerative potential put DPSCs at an advantage compared to BMMSCs for tissue regenerative applications.^74^

Muscle regeneration is another promising application for dental tissue-derived MSCs. In this context, DPSCs have shown promising outcomes.^75^ However, isolation of DPSCs requires tooth extraction, which has limited their application. Alternatively, GMSCs can be used as a potent source with less invasive harvesting procedures. Alginate hydrogels can be used as a platform to encapsulate GMSCs and a cocktail of multiple growth factors (e.g., Forskolin, 6-Bromo-1-methylindirubin-3’-oxime, and basic-FGF) for myogenic differentiation.^76^

Originating from the neural crest, dental MSCs have brought new hopes to neural tissue engineering.^77,78^ It has been reported that almost all types of dental MSCs have the capability of expressing neurotrophic factors including brain‐derived neurotrophic factor (BDNF), glial cell‐derived neurotrophic factor (GDNF), and nerve growth factor (NGF), and can promote the growth of Schwann cells and neurite outgrowths.^79^ In particular, besides the neural differentiation capacity, DPSCs and SHEDs could be a promising candidate for the treatment of spinal cord injuries and neurodegenerative diseases such as Alzheimer’s disease (AD) since they can prevent apoptotic damages to the injured neural cells, hinder the expression of axon growth inhibitors, and promote survival of microglia under neurotoxic conditions by their secretome specifically fractalkine and RANTES.^80,81^

As discussed earlier, the microenvironment plays critical roles in determining the fate of encapsulated MSCs. A properly designed biomaterial with appropriate mechanical properties and incorporated with neurogenic growth factors can secure the survival and neural differentiation of dental MSCs. For instance, an NGF incorporated 3D scaffold based on alginate and hyaluronic acid has shown successful neural differentiation of GMSCs and PDLSc for nerve regeneration, in vitro and in vivo.^82^

Sensorineural hearing loss (SNHL) is a permanent disability that shuts off the individual from the hearing world forever. Scientific progress in this field had been stifled for many years due to anatomical and technical difficulties. Discovering the potential of MSCs in treating SNHL has been a light at the end of tunnel.^83^ In this context, GMSCs, as an easily accessible source of MSCs, have shown promising outcomes in developing auditory progenitor cells when encapsulated within a biomimetic 3D hydrogel and given the necessary growth factor cocktail.^84^

## Biomaterials as a platform for delivery of dental MSCs

The majority of our understanding of many biological processes is based on cellular studies conducted on traditional two-dimensional (2D) substrates. However, 2D systems cannot replicate the natural 3D cell–cell and cell–ECM interactions. A wealth of research showing the limitations of 2D systems highlights the need for novel platforms to mimic the cellular microenvironment as closely as possible.

Naturally, cells reside in a 3D microenvironment where they can establish cell–cell and cell–matrix interactions necessary for normal cellular behavior. However, cell culture plates limit the cells to a 2D surface where they cannot freely form the necessary interactions. Additionally, ex-vivo-expanded cells lose a significant amount of their cellular matrix and cell/matrix interactions upon detachment from the culture plate, which results in a lower survival rate and poor engraftment (~1%) after transplantation in vivo.^85^ The development of 3D systems has emerged as an effective method for mimicking the actual microenvironment of cells more accurately than 2D cell culture systems, meeting the need for a more naturalistic environment in which cells can thrive.

Immune cell invasion and the stress that cells encounter during injection and transplantation are other challenges that lower the survival rate of transplanted cells.^86,87^ A promising way to protect the cells against the immune system and environmental stresses is to encapsulate them within a biomimetic niche-like structure to enhance their survival and thus the success rate of stem cell delivery.^88,89^ Additionally, the 3D culturing of MSCs in the form of an aggregate or within a niche-mimicking biomaterial can manipulate the expression of surface markers, promote cell–cell interactions, enhance sensitivity to the microenvironment, and increase paracrine secretion.^90,91^

Scaffold-based systems can be generated by encapsulating MSCs within a 3D biomaterial in the form of a biodegradable or nonbiodegradable synthetic polymeric scaffold such as poly(ethylene glycol) (PEG), poly(lactic acid) (PLA), poly(lactic-co-glycolic acid) (PLGA), Polymethyl methacrylate (PMMA), Polytetrafluoroethylene (PTFE), Polydimethylsiloxane (PDMS); within a matrix-like hydrogel containing proteins and ECM components such as alginate, collagen, laminin, fibrin, hyaluronic acid, and chitosan followed by solidification or polymerization; or on a biological scaffold composed of a natural acellular 3D matrix such as decellularized tooth buds.^66,92–99^ Some of the widely studied biomaterials are summarized in Table 2.

**Table 2:** Summary of biomaterials used in tissue engineering

Materials	Type	Components
Synthetic material	Biodegradable	Poly(ethylene glycol) (PEG)^173^
		polylactic acid (PLA)^174^
		poly(lactic-co-glycolic acid) (PLGA)^175^
		Gelatin methacrylate (GelMA)^89^
	Nonbiodegradable	Polymethyl methacrylate (PMMA)^176^
		Polytetrafluoroethylene (PTFE)^177^
		Polydimethylsiloxane (PDMS)^178^
Natural material	Hydrogel	Alginate^179^
		Collagen^180,181^
		Laminin^182^
		Fibrin^183^
		Hyaluronic acid^109^
		Chitosan^117^
	Decellularized biological scaffolds	Decellularized Tooth Bud Scaffolds^66^

ECM remodeling is crucial for achieving tissue homeostasis and normal cell behavior; thus, it is important to mimic compositional as well as architectural characteristics of natural ECM in vitro.^100,101^ Many different biomaterials and fabrication techniques have been developed to fabricate scaffolds with appropriate physical and biological characteristics of specific natural microenvironments to fulfill the requirements of different cell types in vitro and in vivo. The features and properties of biomaterials, including their degradation kinetics, molecular compatibility, and porosity, can be tuned to enable spatial and temporal control over the extracellular cues presented to cells.^102,103^

### Hydrogels

A wide range of biomaterials has been studied in the quest to mimic the natural microenvironment of MSCs as closely as possible for optimal tissue regeneration. Hydrogels are among the most deeply investigated biomaterials due to their flexible physiomechanical properties and excellent biocompatibility stemming from their similarity to the macromolecular components in the body.^104,105^ Hydrogels can be comprised of crosslinked polymer chains or complex protein molecules with a natural or synthetic origin. Natural hydrogels are inherently biocompatible and bioactive.^106,107^

Hydrogels’ great potential to mimic the ECM and ability to provide gas and nutrient exchange allows for various clinical applications.^5,108^ They can be engineered to represent the natural extracellular environment of different tissues. Additionally, hydrogels can be combined with stem cells to form an injectable product that can be administered in a minimally invasive way.^109,110^ These unique features have prompted scientists to actively look for revolutionary strategies and new possibilities for their use.^111^

One contemporary strategy would be the application of dental MSC-laden hydrogels for regenerative endodontic purposes. This approach aims to utilize biomaterials for the delivery of dental MSCs to the root canal to reconstruct the pulp-dentin complex and support root development.^112^ For instance, fibrin hydrogel incorporating clindamycin-loaded Poly (D, L) Lactic Acid (PLA) nanoparticles can serve as an antibacterial and antibiofilm platform for the regeneration of devitalized dental pulp.^113^ Alternatively, polyethylene glycol diacrylate (PEGDA)-based biomaterials can be used as injectable hydrogels in which to deliver DPSCs into the root canal lumen.^114^

Chitosan is the second most abundant natural semi-crystalline polysaccharide, derived from the shells of marine crustaceans, insects, or fungi. It is widely used in numerous tissue engineering applications including periodontal tissue regeneration.^115,116^ Chitosan-based injectable hydrogels can be used as a delivery platform for the local release of drugs such as antibiotics and antiseptics to prevent infection and inflammation associated with periodontitis, to release growth factors such bone morphogenetic protein-7 (BMP-7) and basic fibroblast growth factor (bFGF) to stimulate regeneration of lost tissue or to deliver MSCs for periodontal tissue reconstruction.^117–121^

Despite the wide range of studies that have been performed to date, there appears to be a perception that current hydrogels do not remain at a defect site long enough to complete their tissue regenerative mission.^122,123^ They may have extended applications if they can adhere and remain at the defect site in the presence of body fluids such as blood and saliva. This feature is especially significant in cases of oral and craniofacial defects. Polysaccharide-based hydrogels have been the material of choice for numerous tissue engineering studies, but in this regard, their weak adhesion to the biological tissues has limited their application.^124^

**Fig. 3:**
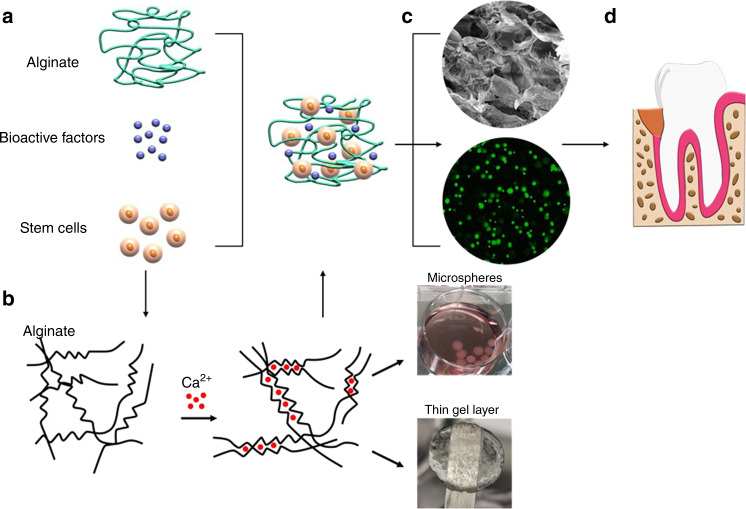
Schematic image demonstrating the steps to develop a cell-laden alginate hydrogel. **a** Alginate solution can be mixed with bioactive factors and the desired source of stem cells before crosslinking. **b** Alginate structure can be crosslinked with the addition of divalent cations such as Ca^2+^. The source of the divalent cation is one of the factors controlling the gelation rate to fabricate different types of alginate hydrogel including microspheres or thin gel layers. **c** The SEM image (upper panel) shows the homogeneous microporous structure, and the live/dead cell viability assay (lower panel) demonstrates higher biocompatibility of the alginate hydrogels. **d** The developed cell-laden hydrogel can be easily administered into the defect site

Alginate is a naturally occurring polysaccharide and a block copolymer composed of (1–4)-linked β-D-mannuronic acid (M blocks) and α-L-guluronic acid (G blocks) monomers and can be used as a stem cells or bioactive factors delivery vehicle (Fig. 3a).^125^ Alginate hydrogels can be developed by crosslinking the solution with a divalent cation such as Ca^2+^, which binds the G blocks and M blocks together. The source of the Ca^2+^ determines the rate of crosslinking reactions.^126^ For instance, calcium sulfate (CaSO_4_) slows the crosslinking reaction due to its lower solubility and provides us with a longer working time to handle and cast the alginate in the form of thin layers (Fig. 3b). In contrast, calcium chloride (CaCl_2_) results in rapid gelation, therefore, is a good alternative to produce alginate microspheres by adding the alginate solution into the CaCl_2_ bath dropwise (Fig. 3b).

Selected sources of MSCs and bioactive factors such as growth factors can be mixed with the alginate solution before crosslinking to induce the desired differentiation of the encapsulated MSCs. As the scanning electron microscopy (SEM) image shows in Fig. 3c (upper panel), alginate hydrogels can provide a homogenous porous microstructure. Additionally, the live/dead assay conducted on the alginate hydrogel encapsulating MSCs is shown in Fig. 3c, lower panel, which confirms its optimal biocompatibility. The cell-laden alginate hydrogels can be easily applied into the defect site (Fig. 3d).

One pioneering study has developed a novel mussel-inspired hydrogel based on alginate with the capability of strongly adhering to wet and dry surfaces as a GMSC delivery vehicle for craniofacial bone regeneration.^127^ This promising material was developed by modifying a methacrylated alginate hydrogel with dopamine to produce a visible-light-crosslinkable adhesive hydrogel with adjustable mechanical properties, which was further loaded with hydroxyapatite microparticles to induce osteogenic differentiation of the encapsulated GMSCs. In vitro studies followed by in vivo studies in rats have confirmed the efficacy of this approach as a promising platform for craniofacial tissue regeneration.

### Biological scaffolds

The concept of growing complex 3D tissues that perfectly mimic the design and function of actual human tissue has emerged recently.^128^ Advances in cellular and molecular biology have opened a window of opportunity for tissue engineering approaches involving decellularized (also called acellular) native tissue as a substitute for artificial scaffolds. Decellularization of any given tissue entails the isolation of components of the native ECM with minimal structural and compositional disruption while discharging the entirety of the cellular contents. These scaffolds preserve the natural architecture of the target organ at the micro- and nano-structural levels and also possess suitable mechanical and biochemical characteristics for recellularization with selected stem cells or other cell populations to induce differentiation towards a target tissue.^129^

Application of decellularized scaffolds for bioengineering has mainly focused on cardiovascular tissue, heart valves, liver, kidney, skin, diabetic foot ulcers, and pancreatic tissue, with little attention paid to sensory organs or dental tissues.^130–134^ However, an innovative milestone has been achieved by Santi et al.,^135,136^ who developed a decellularized cochlea (DC) as a superior 3D scaffold for directing stem cells toward an auditory lineage. They removed ear tissues from euthanized mice, a rat, and a human and decellularized them using two different detergent extraction methods, the strong anionic detergent sodium dodecyl sulfate (SDS) and the weaker anionic detergent sodium deoxycholate (SDOC). Scanning thin-sheet laser imaging microscopy and bright-field microscopy revealed that SDS detergent was more successful than SDOC detergent in extracting cellular elements. Stem cells seem to hold promise as the ideal regenerative cells to be grown and directed toward an auditory phenotype on this decellularized cochlear tissue.

Similar approaches are garnering increased attention in regenerative dentistry, specifically for regenerating nonvital dental pulp. One such study has attempted to decellularize the whole structure of human dental pulp with a low concentration (0.03%) of SDS for use as a biological scaffold.^137^ Their decellularization protocol resulted in the development of a porous acellular scaffold preserving the histoarchitecture and composition of the native tissue including a network of collagen fibers. The decellularized dental pulp tissues could be used as an alternative to conventional root canal techniques to induce cellular infiltration for pulp revitalization.^138,139^

Ultimately, regenerating complete teeth will be a game-changer in modern dentistry. It has been reported that decellularized tooth buds can provide an appropriate platform for differentiation of dental MSCs to generate a whole tooth with organized dentin and enamel-like structures.^66^

## Biomaterials as de novo niches for homing of endogenous MSCs

Currently, the majority of studies aiming towards craniofacial tissue regeneration have mostly relied on transplantation of exogenously manipulated stem cells, which has multiple technical and safety challenges. Despite best efforts, a large fraction of the implanted cells is lost within a few hours of implantation due to various environmental stresses encountered during transplantation and maintenance in the defect site. Thus, a new paradigm for the treatment of damaged tissue is to harness the endogenous healing capability of the local cells residing in the postnatal oral tissue to avoid the injection of exogenously manipulated cells.^140,141^ This paradigm shift could introduce a novel treatment modality for the regeneration of craniofacial/orofacial bone defects by harnessing the physiological healing cascade through recruiting the local MSCs while inducing in situ reprogramming with a flexible scaffold as an artificial niche with tunable mechanical and degradation properties. In such an approach, biomaterials can be used as a platform to stimulate endogenous stem cells.^88^

Developing a practical approach for capitalizing on the regenerative potential of the endogenous cells can overcome the limitations of currently available regenerative medicine strategies with a controlled and safe in vivo lineage reprogramming approach. However, engineering an artificial niche requires a combination of physical and biological characteristics including tunable mechanical properties, an appropriate degradation profile, and the expression of necessary bioactive mediators.

The development of artificial niches offers new strategies for directing in vivo reprogramming by recapitulating salient features of complex biological systems through exhibiting physical, topographical, or biochemical cues.^142,143^ Biomaterials could be exploited as modular toolboxes to construct simplified de novo niches that stimulate the body’s repair mechanisms through the recruitment of endogenous cells by employing bioactive molecules.^144,145^ The physiochemical properties of the artificial niche created in such a manner can be finely tuned to directly reprogram the localized endogenous cells. An ideal biomaterial for such a purpose would be biocompatible and biodegradable with no risk of disease transmission while functionally guiding the necessary biological processes.

In nature, the secretion of paracrine signaling factors such as growth factors, cytokines, and chemokines within a microenvironmental niche helps to maintain homeostasis and tissue architecture.^146^ In addition to their ability to secrete these factors, dental-derived MSCs are widely known for their profound immunoregulatory potential.^56,147^ The recruited MSCs could actively contribute to the cellular niche and induce a local pro-regenerative microenvironment not only by continuously supplying growth factors but also by downregulating immune responses and reducing inflammation in the engineered niche at the transplant site.

**Fig. 4:**
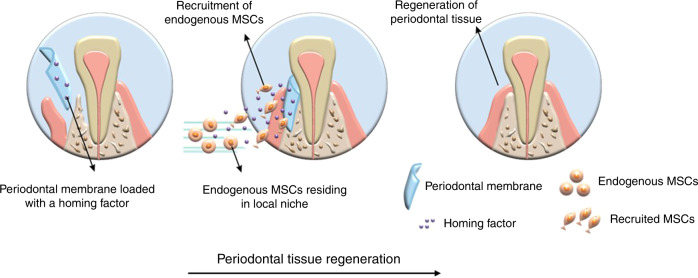
Periodontal membrane as a de novo niche for homing of endogenous MSCs. Chemoattractants can be incorporated into the membrane to induce directional migration of the MSCs to reconstruct the periodontal tissue

Chemoattractants are crucial for recruiting endogenous MSCs for in situ tissue regeneration. Directional migration of the MSCs toward a target site occurs in response to a gradient of soluble chemoattractants through a process called chemotaxis (Fig. 4).^148^ Stem cell factor (SCF) is a potent chemokine known to induce homing of dental pulp progenitor cells for the regeneration of dental pulp.^149^ Furthermore, it supports pulp regeneration by facilitating local cell homing in the pulpless immature root canal.^150^

After an injury, cells local to the injury site strongly express stromal cell-derived factor-1 (SDF-1).^151^ However, the endogenous expression of SDF-1 is short-lasting and might not last long enough to recruit an adequate number of cells to regenerate major defects. This phenomenon has prompted scientists to investigate the functionality of biomaterials incorporating SDF-1 as an artificial niche to recruit local MSCs.^152^ The combination of SDF-1 with biomaterials is one of the most widely studied approaches to recruit different types of endogenous stem cells. For instance, it can be loaded into polyelectrolyte complex nanoparticles and encapsulated within gelatin hydroxyphenyl propionic acid hydrogels to be injected into brain lesions for recruiting local neural progenitor cells.^153^ Similarly, SDF-1 can be released from poly(lactide ethylene oxide fumarate) (PLEOF) hydrogels for recruitment of BMMSCs.^154^ Similar homing activity has been witnessed by the adipose derived stem cells (ADSCs) in response to the release of SDF-1 from an injectable thermoresponsive hydrogel based on chitosan/β-glycerophosphate disodium salt pentahydrate (βGP).^155^

There is enough evidence to accept that adult stem cells are similar in many ways including homing, although some differences might exist among species, and dental-derived MSCs are no exceptions. Despite their outstanding regenerative potential, the fact that tooth extraction and pulp extirpation are needed to harvest DPSCs has limited their clinical translation.^156^ In situ cell homing has emerged in regenerative endodontics research as a new method by which to revitalize necrotic pulp without transplanting exogenous DPSCs.^157^ Similar to its effect on many other types of MSCs, SDF-1 is known to induce migration of DPSCs by optimizing focal adhesion formation accompanied by autophagy.^158^ SDF-1 incorporated into a silk fibroin scaffold has been shown to promote pulp regeneration by inducing DPSC homing in a pulpectomized mature canine preclinical model.^158^ SDF-1 can also induce recruitment and transmigration of SCAP from the apex to the root canal space for regeneration of pulp-dentin structure.^159^

Besides pulp regeneration, the idea of cell homing can be applied to the regeneration of the whole tooth. In one such study, a 3D-printed incisor scaffold was created from poly-ε-caprolactone and hydroxyapatite with interconnecting microchannels loaded with SDF-1 and BMP-7.^160^ After 9 weeks of orthotopic transplantation at a mandibular incisor extraction site, the chemokine-loaded scaffolds could attract a significant number of local cells for regeneration of tooth-like tissue and formation of PDL with periodontal integration.

Granulocyte colony-stimulating factor (G-CSF) is a widely studied cytokine in the mobilization of hematopoietic stem cells (HSCs) or BMMSCs.^161^ However, recruitment of the endogenous MSCs is not generally sufficient to regenerate the defective tissue. In this context, a combinatorial therapy capable of both recruiting and differentiating the MSCs would be highly promising. For instance, it has been shown that (G-CSF) or fibroblast growth factor 2 (FGF-2) has a maximal effect on the migration of SCAPs; however, combining the G-CSF with TGF-β1 could significantly induce both migration and biomineralization of the endogenous SCAPs for regenerative endodontic procedures.^162^ Aside from SCAPs, the G-CSF has shown stimulatory effects on the mobilization of DPSCs from mature teeth. These mobilized DPSCs have shown better vasculogenesis and pulp regeneration compared to the colony-derived DPSCs.^163^

Despite all the promising outcomes, some adverse side effects associated with administration of G-CSF such as altering the HSC niche and osteogenic activity, possible splenic rupture, and myocardial infarction require the development of alternative approaches.^164^ Small molecules could be favorable alternatives in this regard to recruiting endogenous stem cells. Inhibiting the cell adhesion molecules such as VCAM/VLA4 with small molecules has been shown to mobilize hematopoietic stem cells (HSCs).^165^ Natalizumab is an FDA-approved immunosuppressive drug for the treatment of multiple sclerosis and Crohn’s disease and an antibody against VLA4 with potential application in mobilizing the hematopoietic progenitor cells.^166^ AMD3100, a chemokine (C-X-C motif) receptor 4 (CXCR4) antagonist, has also demonstrated successful mobilization of HSCs.^167^

Combining small molecules with chemoattractants has been shown to boost chemoattraction. For instance, combining natalizumab with AMD3100 has been reported to enhance stem cell mobilization.^168^ Simvastatin, a competitive 3-hydroxy-3-methyl coenzyme A (HMG-CoA) reductase inhibitor, has been shown to boost the chemotactic activity of SDF-1 when released from a cell-free PLGA scaffold and ultimately increase migration and bone regenerative potential of endogenous ADSCs.^169^

Manipulating signaling pathways such as Wnt signaling, the Sonic hedgehog (SHH), and Notch signaling pathways are known to increase the stem cell pool.^170^ For instance, activating the SHH pathway with a topical hedgehog agonist application has shown to induce follicular cycling and hair regrowth in adult mouse skin.^171^ Alas, a stark contrast resides in the manipulation of these signaling pathways for regenerative medicine purposes: overactivation of such pathways could result in the formation of tumors.^170^ This concern has lowered the interest in exploring the potential of small molecules in regenerative medicine as an activator (agonist). Additionally, it is not clear if activating these pathways is as easy as inhibiting them intrinsically.

Although in situ recruitment of endogenous MSCs might sound promising, the paucity of endogenous cells at the defect site, specifically in the case of severe defects such as necrotic pulp, and the limited in vivo functionality and short half-life of the administered bioactive factors are among the drawbacks that may limit the clinical translation of this novel approach.^172^ Further studies are needed to develop strategies to overcome these limitations associated for improved tissue regeneration with local cell recruitment.

## Conclusion and future prospects

The craniofacial and orofacial regions’ complex physiological structures and sophisticated tissue architecture have posed serious challenges for conventional methods for regeneration of lost or defective tissues. However, stem-cell-mediated therapeutic interventions have made remarkable progress in recent years, enabling the treatment of diseases that could not be treated by a conventional method. The ideal cell source for widespread clinical adoption would produce autologous stem cells that are easily accessible and abundant, desiderata that may be fulfilled by dental tissue-derived MSCs. Despite the wide range of foundational studies conducted to date, there remains an unmet need for successful regeneration of tissues with stem cell transplantation. Additionally, in the case of successful translation, the level of evidence for tissue regeneration remains limited to sporadic case reports and is not strong enough to be recommended for most clinical applications. Stem cell transplantation is promising but cannot yet be considered a panacea. The concept of in situ tissue regeneration with the recruitment of local cells, rather than cell delivery, may accelerate clinical translation. However, the drawbacks associated with this strategy necessitate further investigation before translation into the clinic for widespread clinical adoption.

## References

[1] Goss R. J. *Principles of Regeneration* (Academic Press, 1969).

[2] Frohlich M (2008). Tissue engineered bone grafts: biological requirements, tissue culture and clinical relevance. Curr. Stem Cell Res. Ther..

[3] Langer R, Vacanti JP (1993). Tissue engineering. Science.

[4] Zhang Z, Gupte MJ, Ma PX (2013). Biomaterials and stem cells for tissue engineering. Expert. Opin. Biol. Ther..

[5] Zahedi, E., Ansari, S., Wu, B. M., Bencharitm, S. & Moshaverinia A. Hydrogels in craniofacial tissue engineering. *Biomater. Oral Dent. Tissue Eng*. 10.1016/B978-0-08-100961-1.00004-9 (2017).

[6] Harris DT (2014). Stem cell banking for regenerative and personalized medicine. Biomedicines.

[7] Polak, J. M. & Bishop, A. E. Stem cells and tissue engineering: past, present, and future. *Ann. N Y Acad. Sci*. 10.1196/annals.1346.001 (2006). 10.1196/annals.1346.001 16831937

[8] Ghosh D, Mehta N, Patil A, Sengupta J (2016). Ethical issues in biomedical use of human embryonic stem cells (hESCs). J. Reprod. Heal. Med.

[9] Shilpa P, Sultana N, Kaul R, Bhat S (2013). Stem cells: boon to dentistry and medicine. Dent. Res. J. (Isfahan).

[10] Marion NW, Mao JJ (2006). Mesenchymal stem cells and tissue engineering. Methods Enzymol..

[11] Dominici M (2006). Minimal criteria for defining multipotent mesenchymal stromal cells. The International Society for Cellular Therapy position statement. Cytotherapy.

[12] Lin H, Sohn J, Shen H, Langhans MT, Tuan RS (2019). Bone marrow mesenchymal stem cells: aging and tissue engineering applications to enhance bone healing. Biomaterials.

[13] Zhou X, Hong Y, Zhang H, Li X (2020). Mesenchymal stem cell senescence and rejuvenation: current status and challenges. Front. Cell Dev. Biol..

[14] Huang GTJ, Gronthos S, Shi S (2009). Mesenchymal stem cells derived from dental tissues vs. those from other sources: their biology and role in Regenerative Medicine. J. Dent. Res..

[15] Ansari S (2017). Dental and orofacial mesenchymal stem cells in craniofacial regeneration: the prosthodontist’s point of view. J. Prosthet. Dent..

[16] Wang H (2018). Comparative characterization of SHED and DPSCs during extended cultivation in vitro. Mol. Med. Rep..

[17] Ansari S (2016). Muscle tissue engineering using gingival mesenchymal stem cells encapsulated in alginate hydrogels containing multiple growth factors. Ann. Biomed. Eng..

[18] Santanna J, Fernandez TL, Bueno DF, Pinheiro CC, Hernandez AJ (2020). Cartilage regeneration with human dental pulp stem cells—a systematic review. Cytotherapy.

[19] Sevari SP (2020). Bioactive glass-containing hydrogel delivery system for osteogenic differentiation of human dental pulp stem cells. J. Biomed. Mater. Res. Part A.

[20] Tomasello, L. et al. Mesenchymal stem cells derived from inflamed dental pulpal and gingival tissue: a potential application for bone formation. *Stem Cell Res. Ther*. 10.1186/s13287-017-0633-z (2017). 10.1186/s13287-017-0633-z PMC5540218 28764802

[21] Ledesma-Martínez, E., Mendoza-Núñez, V. M. & Santiago-Osorio, E. Mesenchymal stem cells derived from dental pulp: a review. *Stem Cells Int*. 10.1155/2016/4709572 (2016). 10.1155/2016/4709572 PMC4686712 26779263

[22] Liang Z (2018). Minced pulp as source of pulpal mesenchymal stem cells with odontogenic differentiation capacity. J. Endod..

[23] Nada OA, El Backly RM (2018). Stem cells from the apical papilla (SCAP) as a tool for endogenous tissue regeneration. Front. Bioeng. Biotechnol..

[24] Huang GTJ (2011). Dental pulp and dentin tissue engineering and regeneration: advancement and challenge. Front. Biosci. Elit.

[25] Zhang J (2019). Dental follicle stem cells: tissue engineering and immunomodulation. Stem Cells Dev..

[26] Morsczeck C (2005). Isolation of precursor cells (PCs) from human dental follicle of wisdom teeth. Matrix Biol..

[27] Nagatomo K (2006). Stem cell properties of human periodontal ligament cells. J. Periodontal Res.

[28] Gay IC, Chen S, MacDougall M (2007). Isolation and characterization of multipotent human periodontal ligament stem cells. Orthod. Craniofacial Res..

[29] Venkatesh D, Kumar KPM, Alur JB (2017). Gingival mesenchymal stem cells. J. Oral. Maxillofac. Pathol..

[30] Fawzy El-Sayed KM, Dörfer CE (2016). Gingival mesenchymal stem/progenitor cells: a unique tissue engineering gem. Stem Cells Int..

[31] Pihlstrom, B. L., Michalowicz, B. S. & Johnson, N. W. Periodontal diseases. *Lancet*10.1016/S0140-6736(05)67728-8 (2005). 10.1016/S0140-6736(05)67728-8 16298220

[32] Sage PT (2012). Antigen recognition is facilitated by invadosome-like protrusions formed by memory/effector T cells. J. Immunol..

[33] Moshaverinia, A. et al. Encapsulated dental-derived mesenchymal stem cells in an injectable and biodegradable scaffold for applications in bone tissue engineering. *J. Biomed. Mater. Res. Part A*10.1002/jbm.a.34546 (2013). 10.1002/jbm.a.34546 23983201

[34] Nagata M (2017). Conditioned medium from periodontal ligament stem cells enhances periodontal regeneration. Tissue Eng. Part A.

[35] Gao X (2018). Immunomodulatory role of stem cells from human exfoliated deciduous teeth on periodontal regeneration. Tissue Eng. Part A.

[36] Raju R (2020). Three-dimensional periodontal tissue regeneration using a bone-ligament complex cell sheet. Sci. Rep..

[37] Bottino MC, Thomas V (2015). Membranes for periodontal regeneration—A materials perspective. Front. Oral. Biol..

[38] Bottino MC (2012). Recent advances in the development of GTR/GBR membranes for periodontal regeneration—a materials perspective. Dent. Mater..

[39] Sam G, Pillai BRM (2014). Evolution of barrier membranes in periodontal regeneration—"Are the third generation membranes really here?". J. Clin. Diagn. Res..

[40] Tamayol A (2013). Fiber-based tissue engineering: progress, challenges, and opportunities. Biotechnol. Adv..

[41] Lannutti J, Reneker D, Ma T, Tomasko D, Farson D (2007). Electrospinning for tissue engineering scaffolds. Mater. Sci. Eng. C..

[42] Huang ZM, Zhang YZ, Kotaki M, Ramakrishna S (2003). A review on polymer nanofibers by electrospinning and their applications in nanocomposites. Compos. Sci. Technol..

[43] Wade RJ, Bassin EJ, Rodell CB, Burdick JA (2015). Protease-degradable electrospun fibrous hydrogels. Nat. Commun..

[44] Bottino MC (2012). Recent advances in the development of GTR/GBR membranes for periodontal regeneration—a materials perspective. Dent. Mater..

[45] Osathanon, T., Chanjavanakul, P., Kongdecha, P., Clayhan, P. & Huynh, N. C.-N. Polycaprolactone-based biomaterials for guided tissue regeneration membrane. *Periodontitis*10.5772/intechopen.69153 (2017).

[46] Hasani-Sadrabadi MM (2019). Hierarchically patterned polydopamine-containing membranes for periodontal tissue engineering. ACS Nano.

[47] Ryu J, Ku SH, Lee H, Park CB (2010). Mussel-inspired polydopamine coating as a universal route to hydroxyapatite crystallization. Adv. Funct. Mater..

[48] Kord Forooshani P (2019). Antibacterial properties of mussel-inspired polydopamine coatings prepared by a simple two-step shaking-assisted method. Front. Chem..

[49] Ercal, P. & Pekozer, G. G. A current overview of scaffold-based bone regeneration strategies with dental stem cells. *Adv. Exp. Med. Biol*. 10.1007/5584_2020_505 (2020). 10.1007/5584_2020_505 32185698

[50] Luong LN, Ramaswamy J, Kohn DH (2012). Effects of osteogenic growth factors on bone marrow stromal cell differentiation in a mineral-based delivery system. Biomaterials.

[51] Trubiani O (2019). Human oral stem cells, biomaterials and extracellular vesicles: a promising tool in bone tissue repair. Int. J. Mol. Sci..

[52] Nakajima K (2018). Comparison of the bone regeneration ability between stem cells from human exfoliated deciduous teeth, human dental pulp stem cells and human bone marrow mesenchymal stem cells. Biochem. Biophys. Res. Commun..

[53] Zheng C, Chen J, Liu S, Jin Y (2019). Stem cell-based bone and dental regeneration: a view of microenvironmental modulation. Int. J. Oral. Sci..

[54] Liu Y (2011). Mesenchymal stem cell-based tissue regeneration is governed by recipient T lymphocytes via IFN-γ and TNF-α. Nat. Med..

[55] Liao L (2016). TNF-α inhibits FoxO1 by upregulating MIR-705 to aggravate oxidative damage in bone marrow-derived mesenchymal stem cells during osteoporosis. Stem Cells.

[56] Moshaverinia A (2015). Regulation of the stem cell-host immune system interplay using hydrogel coencapsulation system with an anti-inflammatory drug. Adv. Funct. Mater..

[57] Moshaverinia A (2013). Co-encapsulation of anti-BMP2 monoclonal antibody and mesenchymal stem cells in alginate microspheres for bone tissue engineering. Biomaterials.

[58] Moshaverinia, A. et al. Bone regeneration potential of stem cells derived from periodontal ligament or gingival tissue sources encapsulated in RGD-modified alginate scaffold. *Tissue Eng. Part A*10.1089/ten.tea.2013.0229 (2013). 10.1089/ten.tea.2013.0229 PMC3926152 24070211

[59] Moya A (2018). Human mesenchymal stem cell failure to adapt to glucose shortage and rapidly use intracellular energy reserves through glycolysis explains poor cell survival after implantation. Stem Cells.

[60] Pirosa, A., Gottardi, R., Alexander, P. G. & Tuan, R. S. Engineering in-vitro stem cell-based vascularized bone models for drug screening and predictive toxicology. *Stem Cell Res. Ther*. 10.1186/s13287-018-0847-8 (2018). 10.1186/s13287-018-0847-8 PMC5910611 29678192

[61] Ercal P, Pekozer GG, Kose GT (2018). Dental stem cells in bone tissue engineering: current overview and challenges. Adv. Exp. Med. Biol.

[62] Hanif A, Qureshi S, Sheikh Z, Rashid H (2017). Complications in implant dentistry. Eur. J. Dent..

[63] Yelick PC, Sharpe PT (2019). Tooth bioengineering and regenerative dentistry. J. Dent. Res..

[64] Oshima, M. & Tsuji, T. Whole tooth regeneration as a future dental treatment. *Adv. Exp. Med. Biol*. 10.1007/978-3-319-22345-2_14 (2015). 10.1007/978-3-319-22345-2_14 26545754

[65] Cheng, N., Wen, J., Hitching, R., Lei, C. & Xu, C. In *Regenerative Approaches in Dentistry*, 89–102 (Springer International Publishing, 2021)

[66] Zhang W, Vazquez B, Oreadi D, Yelick PC (2017). Decellularized tooth bud scaffolds for tooth regeneration. J. Dent. Res..

[67] Smith E. E., Yelick P. C. Bioengineering tooth bud constructs using GelMA hydrogel. *Methods Mol. Biol*. 10.1007/978-1-4939-9012-2_14 (2019). 10.1007/978-1-4939-9012-2_14 PMC6685533 30838572

[68] Duailibi MT (2004). Bioengineered teeth from cultured rat tooth bud cells. J. Dent. Res..

[69] Sonoyama, W. et al. Mesenchymal stem cell-mediated functional tooth regeneration in Swine. *PLoS ONE*10.1371/journal.pone.0000079 (2006) 10.1371/journal.pone.0000079 PMC1762318 17183711

[70] Kim K, Lee CH, Kim BK, Mao JJ (2010). Anatomically shaped tooth and periodontal regeneration by cell homing. J. Dent. Res..

[71] Moshaverinia A (2013). Dental mesenchymal stem cells encapsulated in an alginate hydrogel co-delivery microencapsulation system for cartilage regeneration. Acta Biomater..

[72] Fernandes TL (2020). Systematic review of human dental pulp stem cells for cartilage regeneration. Tissue Eng. Part B Rev..

[73] Satué, M., Schüler, C., Ginner, N. & Erben, R. G. Intra-articularly injected mesenchymal stem cells promote cartilage regeneration, but do not permanently engraft in distant organs. *Sci Rep*. 10.1038/s41598-019-46554-5 (2019). 10.1038/s41598-019-46554-5 PMC6626061 31300685

[74] Ishizaka R (2013). Stimulation of angiogenesis, neurogenesis and regeneration by side population cells from dental pulp. Biomaterials.

[75] Martínez-Sarrà E (2017). Human dental pulp pluripotent-like stem cells promote wound healing and muscle regeneration. Stem Cell Res. Ther..

[76] Ansari S (2016). Muscle Tissue engineering using gingival mesenchymal stem cells encapsulated in alginate hydrogels containing multiple growth factors. Ann. Biomed. Eng..

[77] Ibarretxe G (2012). Neural crest stem cells from dental tissues: a new hope for dental and neural regeneration. Stem Cells Int.

[78] Sharpe PT (2016). Dental mesenchymal stem cells. Dev.

[79] Kolar MK (2017). The neurotrophic effects of different human dental mesenchymal stem cells. Sci. Rep..

[80] Sakai K (2012). Human dental pulp-derived stem cells promote locomotor recovery after complete transection of the rat spinal cord by multiple neuro-regenerative mechanisms. J. Clin. Invest..

[81] NE-MB Ahmed, Murakami M, Hirose Y, Nakashima M (2016). Therapeutic potential of dental pulp stem cell secretome for alzheimer’s disease treatment: an in vitro study. Stem Cells Int.

[82] Ansari, S. et al. Human periodontal ligament- and gingiva-derived mesenchymal stem cells promote nerve regeneration when encapsulated in alginate/hyaluronic acid 3D scaffold. *Adv. Healthc. Mater*. 10.1002/adhm.201700670 (2017). 10.1002/adhm.201700670 PMC5813692 29076281

[83] Chorath KT, Willis MJ, Morton-Gonzaba N, Humann WJ, Moreira A (2019). Mesenchymal stem cells for sensorineural hearing loss: protocol for a systematic review of preclinical studies. Syst. Rev..

[84] Pouraghaei S, Moztarzadeh F, Chen C, Ansari S, Moshaverinia A (2020). Microenvironment can induce development of auditory progenitor cells from human gingival mesenchymal stem cells. ACS Biomater. Sci. Eng..

[85] Kean TJ, Lin P, Caplan AI, Dennis JE (2013). MSCs: delivery routes and engraftment, cell-targeting strategies, and immune modulation. Stem Cells Int.

[86] Lukomska, B. et al. Challenges and controversies in human mesenchymal stem cell therapy. *Stem Cells Int*. 10.1155/2019/9628536 (2019). 10.1155/2019/9628536 PMC6481040 31093291

[87] Ansari S (2017). Hydrogel elasticity and microarchitecture regulate dental-derived mesenchymal stem cell-host immune system cross-talk. Acta Biomater..

[88] Peerani R, Zandstra PW (2010). Enabling stem cell therapies through synthetic stem cell-niche engineering. J. Clin. Invest..

[89] Ansari S (2017). Regulation of the fate of dental-derived mesenchymal stem cells using engineered alginate-GelMA hydrogels. J. Biomed. Mater. Res. Part A.

[90] Wang X, Ye K, Li Z, Yan C, Ding J (2013). Adhesion, proliferation, and differentiation of mesenchymal stem cells on RGD nanopatterns of varied nanospacings. Organogenesis.

[91] Follin B (2016). Increased paracrine immunomodulatory potential of mesenchymal stromal cells in three-dimensional culture. Tissue Eng. Part B Rev..

[92] McKinnon DD, Kloxin AM, Anseth KS (2013). Synthetic hydrogel platform for three-dimensional culture of embryonic stem cell-derived motor neurons. Biomater. Sci..

[93] Natesan S, Zamora DO, Suggs LJ, Christy RJ (2012). Engineering a bilayered hydrogel to control ASC differentiation. J. Vis. Exp..

[94] Spencer NJ, Cotanche DA, Klapperich CM (2008). Peptide- and collagen-based hydrogel substrates for in vitro culture of chick cochleae. Biomaterials.

[95] Carletti, E., Motta, A. & Migliaresi, C. Scaffolds for tissue engineering and 3D cell culture. *Methods Mol. Biol.*10.1007/978-1-60761-984-0_2 (2011). 10.1007/978-1-60761-984-0_2 21042963

[96] Edmondson R, Broglie JJ, Adcock AF, Yang L (2014). Three-dimensional cell culture systems and their applications in drug discovery and cell-based biosensors. Assay. Drug Dev. Technol..

[97] Lee, E., Milan, A., Urbani, L., De Coppi, P. & Lowdell, M. W. Decellularized material as scaffolds for tissue engineering studies in long gap esophageal atresia. *Expert. Opin. Biol. Ther*. 10.1080/14712598.2017.1308482 (2017). 10.1080/14712598.2017.1308482 28303723

[98] Duisit, J. et al. Decellularization of the porcine ear generates a biocompatible, nonimmunogenic extracellular matrix platform for face subunit bioengineering. *Ann. Surg*. 10.1097/SLA.0000000000002181 (2017). 10.1097/SLA.0000000000002181 28252516

[99] Ngoenkam J, Faikrua A, Yasothornsrikul S, Viyoch J (2010). Potential of an injectable chitosan/starch/ˇ-glycerol phosphate hydrogel for sustaining normal chondrocyte function. Int. J. Pharm..

[100] Gattazzo F, Urciuolo A, Bonaldo P (2014). A dynamic microenvironment for stem cell niche. Biochim. Biophys. Acta.

[101] DuFort CC, Paszek MJ, Weaver VM (2011). Balancing forces: architectural control of mechanotransduction. Nat. Rev. Mol. Cell Biol..

[102] Bratt-Leal AM, Carpenedo RL, Ungrin MD, Zandstra PW, McDevitt TC (2011). Incorporation of biomaterials in multicellular aggregates modulates pluripotent stem cell differentiation. Biomaterials.

[103] Sionkowska A (2011). Current research on the blends of natural and synthetic polymers as new biomaterials: Review. Prog. Polym. Sci..

[104] Zhu J, Marchant RE (2011). Design properties of hydrogel tissue-engineering scaffolds. Expert. Rev. Med. Devices.

[105] Jhon MS, Andrade JD (1973). Water and hydrogels. J. Biomed. Mater. Res.

[106] Hoffman AS (2012). Hydrogels for biomedical applications. Adv. Drug Deliv. Rev..

[107] Seliktar D (2012). Designing cell-compatible hydrogels for biomedical applications. Science.

[108] Lee KY, Mooney DJ (2001). Hydrogels for tissue engineering. Chem. Rev..

[109] Ansari S (2017). Alginate/hyaluronic acid hydrogel delivery system characteristics regulate the differentiation of periodontal ligament stem cells toward chondrogenic lineage. J. Mater. Sci. Mater. Med..

[110] Sivashanmugam A, Arun Kumar R, Vishnu Priya M, Nair SV, Jayakumar R (2015). An overview of injectable polymeric hydrogels for tissue engineering. Eur. Polym. J..

[111] Diniz IMA (2016). Gingival mesenchymal stem cell (GMSC) delivery system based on RGD-coupled alginate hydrogel with antimicrobial properties: a novel treatment modality for peri-implantitis. J. Prosthodont.

[112] Raddall G, Mello I, Leung BM (2019). Biomaterials and scaffold design strategies for regenerative endodontic therapy. Front. Bioeng. Biotechnol..

[113] Bekhouche M (2020). Development of an antibacterial nanocomposite hydrogel for human dental pulp engineering. J. Mater. Chem. B.

[114] Jones TD, Kefi A, Sun S, Cho M, Alapati SB (2016). An optimized injectable hydrogel scaffold supports human dental pulp stem cell viability and spreading. Adv. Med..

[115] Croisier F, Jérôme C (2013). Chitosan-based biomaterials for tissue engineering. Eur. Polym. J..

[116] Sultankulov, B., Berillo, D., Sultankulova, K., Tokay, T. & Saparov, A. Progress in the development of chitosan-based biomaterials for tissue engineering and regenerative medicine. *Biomolecules*10.3390/biom9090470 (2019). 10.3390/biom9090470 PMC6770583 31509976

[117] Tang G (2020). Recent advances of chitosan-based injectable hydrogels for bone and dental tissue regeneration. Front. Bioeng. Biotechnol..

[118] Zang S (2019). Injectable chitosan/β-glycerophosphate hydrogels with sustained release of BMP-7 and ornidazole in periodontal wound healing of class III furcation defects. Mater. Sci. Eng. C..

[119] Miranda DG (2016). A chitosan-hyaluronic acid hydrogel scaffold for periodontal tissue engineering. J. Biomed. Mater. Res. Part B Appl. Biomater..

[120] Ouchi T, Nakagawa T (2020). Mesenchymal stem cell-based tissue regeneration therapies for periodontitis. Regen. Ther..

[121] Murakami S (2003). Recombinant human basic fibroblast growth factor (bFGF) stimulates periodontal regeneration in class II furcation defects created in beagle dogs. J. Periodontal Res.

[122] Suneetha M, Rao KM, Han SS (2019). Mussel-inspired cell/tissue-adhesive, hemostatic hydrogels for tissue engineering applications. ACS Omega.

[123] Mehdizadeh M, Yang J (2013). Design strategies and applications of tissue bioadhesives. Macromol. Biosci..

[124] Zhu T (2019). Recent progress of polysaccharide‐based hydrogel interfaces for wound healing and tissue engineering. Adv. Mater. Interfaces.

[125] Rowley JA, Madlambayan G, Mooney DJ (1999). Alginate hydrogels as synthetic extracellular matrix materials. Biomaterials.

[126] Lee KY, Mooney DJ (2012). Alginate: properties and biomedical applications. Prog. Polym. Sci..

[127] Hasani-Sadrabadi MM (2020). An engineered cell-laden adhesive hydrogel promotes craniofacial bone tissue regeneration in rats.. Sci. Transl. Med.

[128] Heidaran mohammad A (2000). Tissue engineering: a biological solution for tissue damage, loss or end stage organ failure. Iran. Biomed. J..

[129] Rana, D., Zreiqat, H., Benkirane-jessel, N., Ramakrishna, S. & Ramalingam, M. Development of decellularized scaffolds for stem cell-driven tissue engineering. *J. Tissue Eng. Regen. Med.*10.1002/term (2017). 10.1002/term.2061 26119160

[130] Tapias LF, Ott HC (2014). Decellularized scaffolds as a platform for bioengineered organs. Curr. Opin. Organ Transpl..

[131] Chamberland C (2014). Embryonic decellularized cardiac scaffold supports embryonic stem cell differentiation to produce beating cardiac tissue. ISRN Stem Cells.

[132] Boccafoschi F (2017). Decellularized biological matrices: an interesting approach for cardiovascular tissue repair and regeneration. J. Tissue Eng. Regen. Med..

[133] Theodoridis K (2015). Successful matrix guided tissue regeneration of decellularized pulmonary heart valve allografts in elderly sheep. Biomaterials.

[134] Mazza, G. et al. Decellularized human liver as a natural 3D-scaffold for liver bioengineering and transplantation. *Nat. Publ. Gr*. 10.1038/srep13079 (2015). 10.1038/srep13079 PMC4528226 26248878

[135] Santi PA, Johnson SB (2013). Decellularized ear tissues as scaffolds for stem cell differentiation. J. Assoc. Res. Otolaryngol..

[136] Santi PA (2016). Scanning electron microscopic examination of the extracellular matrix in the decellularized mouse and human cochlea. J. Assoc. Res. Otolaryngol..

[137] Matoug-Elwerfelli M, Duggal MS, Nazzal H, Esteves F, Raïf E (2018). A biocompatible decellularized pulp scaffold for regenerative endodontics. Int. Endod. J..

[138] Alqahtani Q (2018). Decellularized swine dental pulp tissue for regenerative root canal therapy. J. Dent. Res..

[139] Hu, L. et al. Decellularized swine dental pulp as a bioscaffold for pulp regeneration. *Biomed Res. Int*. 10.1155/2017/9342714 (2017). 10.1155/2017/9342714 PMC5745671 29387727

[140] Dimmeler S, Ding S, Rando TA, Trounson A (2014). Translational strategies and challenges in regenerative medicine. Nat. Med..

[141] Chen FM, Wu LA, Zhang M, Zhang R, Sun HH (2011). Homing of endogenous stem/progenitor cells for in situ tissue regeneration: Promises, strategies, and translational perspectives. Biomaterials.

[142] Kim E, Tae G (2016). Direct reprogramming and biomaterials for controlling cell fate. Biomater. Res..

[143] Lenzini S, Devine D, Shin JW (2019). Leveraging biomaterial mechanics to improve pluripotent stem cell applications for tissue engineering. Front. Bioeng. Biotechnol..

[144] Donnelly, H., Salmeron-Sanchez, M. & Dalby, M. J. Designing stem cell niches for differentiation and self-renewal. *J. R. Soc. Interface*10.1098/rsif.2018.0388 (2018). 10.1098/rsif.2018.0388 PMC6127175 30158185

[145] Lutolf MP, Blau HM (2009). Artificial stem cell niches. Adv. Mater..

[146] Han Y, You X, Xing W, Zhang Z, Zou W (2018). Paracrine and endocrine actions of bone—-the functions of secretory proteins from osteoblasts, osteocytes, and osteoclasts. Bone Res..

[147] Andrukhov O, Behm C, Blufstein A, Rausch-Fan X (2019). Immunomodulatory properties of dental tissue-derived mesenchymal stem cells: Implication in disease and tissue regeneration. World J. Stem Cells.

[148] Vanden Berg-Foels WS (2014). In situ tissue regeneration: chemoattractants for endogenous stem cell recruitment. Tissue Eng. Part B Rev..

[149] Pan S (2013). SCF promotes dental pulp progenitor migration, neovascularization, and collagen remodeling—potential applications as a homing factor in dental pulp regeneration. Stem Cell Rev. Rep..

[150] Ruangsawasdi N (2017). Effects of stem cell factor on cell homing during functional pulp regeneration in human immature teeth. Tissue Eng. Part A.

[151] Lau TT, Wang DA (2011). Stromal cell-derived factor-1 (SDF-1): Homing factor for engineered regenerative medicine. Expert. Opin. Biol. Ther..

[152] Wang J (2019). Incorporation of stromal cell-derived factor-1α in three-dimensional hydroxyapatite/polyacrylonitrile composite scaffolds for bone regeneration. ACS Biomater. Sci. Eng..

[153] Lim TC (2013). Chemotactic recruitment of adult neural progenitor cells into multifunctional hydrogels providing sustained SDF-1α release and compatible structural support. FASEB J..

[154] He X, Ma J, Jabbari E (2010). Migration of marrow stromal cells in response to sustained release of stromal-derived factor-1α from poly(lactide ethylene oxide fumarate) hydrogels. Int. J. Pharm..

[155] Fadera S, Cheng NC, Young TH, Lee IC (2020). In vitro study of SDF-1α-loaded injectable and thermally responsive hydrogels for adipose stem cell therapy by SDF-1/CXCR4 axis. J. Mater. Chem. B.

[156] Mao JJ (2012). Regenerative endodontics. Barriers and strategies for clinical translation. Dent. Clin. North Am..

[157] Eramo S, Natali A, Pinna R, Milia E (2018). Dental pulp regeneration *via* cell homing. Int. Endod. J..

[158] Yang, J. W. et al. Autophagy in SDF-1α-mediated DPSC migration and pulp regeneration. *Biomaterials* **44**, 11–23 (2015). 10.1016/j.biomaterials.2014.12.006 25617122

[159] Liu JY, Chen X, Yue L, Huang GTJ, Zou XY (2015). CXC Chemokine receptor 4 is expressed paravascularly in apical papilla and coordinates with stromal cell-derived factor-1α during transmigration of stem cells from apical papilla. J. Endod..

[160] Kim K, Lee CH, Kim BK, Mao JJ (2010). Anatomically shaped tooth and periodontal regeneration by cell homing. J. Dent. Res.

[161] Wu CC (2017). G-CSF-mobilized bone marrow mesenchymal stem cells replenish neural lineages in alzheimer’s disease mice via CXCR4/SDF-1 chemotaxis. Mol. Neurobiol..

[162] Fayazi S, Takimoto K, Diogenes A (2017). Comparative evaluation of chemotactic factor effect on migration and differentiation of stem cells of the apical papilla. J. Endod..

[163] Nakayama H (2017). Enhanced regeneration potential of mobilized dental pulp stem cells from immature teeth. Oral. Dis..

[164] Winkler IG (2012). Hematopoietic stem cell mobilizing agents G-CSF, cyclophosphamide or AMD3100 have distinct mechanisms of action on bone marrow HSC niches and bone formation. Leukemia.

[165] Xin ZC, Xu YDE, Lin G, Lue TF, Guo YL (2016). Recruiting endogenous stem cells: a novel therapeutic approach for erectile dysfunction. Asian J. Androl..

[166] Zohren F (2008). The monoclonal anti-VLA-4 antibody natalizumab mobilizes CD34 hematopoietic progenitor cells in humans. Blood.

[167] Liles WC (2003). Mobilization of hematopoietic progenitor cells in healthy volunteers by AMD3100, a CXCR4 antagonist. Blood.

[168] Bonig H, Watts KL, Chang K-H, Kiem H-P, Papayannopoulou T (2009). Concurrent blockade of α4-integrin and CXCR4 in hematopoietic stem/progenitor cell mobilization. Stem Cells.

[169] Liu Y-S (2014). The effect of simvastatin on chemotactic capability of SDF-1α and the promotion of bone regeneration. Biomaterials.

[170] Lu B, Atala A (2014). Small molecules and small molecule drugs in regenerative medicine. Drug Discov. Today.

[171] Paladini RD, Saleh J, Qian C, Xu GX, Rubin LL (2005). Modulation of hair growth with small molecule agonists of the hedgehog signaling pathway. J. Invest. Dermatol.

[172] Ullah M, Liu DD, Thakor AS (2019). Mesenchymal stromal cell homing: mechanisms and strategies for improvement. iScience.

[173] Galler KM (2011). Bioengineering of dental stem cells in a PEGylated fibrin gel. Regen. Med..

[174] Diomede F (2018). Three-dimensional printed PLA scaffold and human gingival stem cell-derived extracellular vesicles: A new tool for bone defect repair. Stem Cell Res. Ther..

[175] Gangolli RA, Devlin SM, Gerstenhaber JA, Lelkes PI, Yang M (2019). A bilayered poly (Lactic-Co-Glycolic Acid) scaffold provides differential cues for the differentiation of dental pulp stem cells. Tissue Eng. Part A..

[176] Shirzad M, Matbouei A, Fathi A, Rabiee SM (2020). Experimental and numerical investigation of polymethyl methacrylate scaffolds for bone tissue engineering. Proc. Inst. Mech. Eng. Part L J. Mater. Des. Appl..

[177] Korzinskas T (2018). In vivo analysis of the biocompatibility and macrophage response of a non-resorbable PTFE membrane for guided bone regeneration. Int. J. Mol. Sci..

[178] Yan X, JJJP Beucken, Yuan C, Jansen JA, Yang F (2019). Evaluation of polydimethylsiloxane‐based substrates for in vitro culture of human periodontal ligament cells. J. Biomed. Mater. Res. Part A.

[179] Moshaverinia A (2012). Alginate hydrogel as a promising scaffold for dental-derived stem cells: an in vitro study. J. Mater. Sci. Mater. Med..

[180] Coyac BR (2013). Mineralization of dense collagen hydrogel scaffolds by human pulp cells. J. Dent. Res.

[181] Xie Y (2017). Collagen sponge functionalized with chimeric anti-BMP-2 monoclonal antibody mediates repair of critical-size mandibular continuity defects in a nonhuman primate model. Biomed. Res. Int..

[182] Fu, J. et al. Laminin-modified dental pulp extracellular matrix for dental pulp regeneration. *Front. Bioeng. Biotechnol*. 10.3389/fbioe.2020.595096 (2021). 10.3389/fbioe.2020.595096 PMC7838611 33520954

[183] Parisi L (2020). A glance on the role of fibronectin in controlling cell response at biomaterial interface. Jpn Dent. Sci. Rev..

